# Mental Workload Estimation Based on Physiological Features for Pilot-UAV Teaming Applications

**DOI:** 10.3389/fnhum.2021.692878

**Published:** 2021-08-20

**Authors:** Gaganpreet Singh, Caroline P. C. Chanel, Raphaëlle N. Roy

**Affiliations:** ^1^ISAE-SUPAERO, Université de Toulouse, Toulouse, France; ^2^Artificial and Natural Intelligence Toulouse Institute – ANITI, Toulouse, France

**Keywords:** mental workload, pilot-UAV teaming, EEG, ECG, eye-tracking, ecological classification design

## Abstract

Manned-Unmanned Teaming (MUM-T) can be defined as the teaming of aerial robots (artificial agents) along with a human pilot (natural agent), in which the human agent is not an authoritative controller but rather a cooperative team player. To our knowledge, no study has yet evaluated the impact of MUM-T scenarios on operators' mental workload (MW) using a neuroergonomic approach (i.e., using physiological measures), nor provided a MW estimation through classification applied on those measures. Moreover, the impact of the non-stationarity of the physiological signal is seldom taken into account in classification pipelines, particularly regarding the validation design. Therefore this study was designed with two goals: (i) to characterize and estimate MW in a MUM-T setting based on physiological signals; (ii) to assess the impact of the validation procedure on classification accuracy. In this context, a search and rescue (S&R) scenario was developed in which 14 participants played the role of a pilot cooperating with three UAVs (Unmanned Aerial Vehicles). Missions were designed to induce high and low MW levels, which were evaluated using self-reported, behavioral and physiological measures (i.e., cerebral, cardiac, and oculomotor features). Supervised classification pipelines based on various combinations of these physiological features were benchmarked, and two validation procedures were compared (i.e., a traditional one that does not take time into account vs. an ecological one that does). The main results are: (i) a significant impact of MW on all measures, (ii) a higher intra-subject classification accuracy (75%) reached using ECG features alone or in combination with EEG and ET ones with the Adaboost, Linear Discriminant Analysis or the Support Vector Machine classifiers. However this was only true with the traditional validation. There was a significant drop in classification accuracy using the ecological one. Interestingly, inter-subject classification with ecological validation (59.8%) surpassed both intra-subject with ecological and inter-subject with traditional validation. These results highlight the need for further developments to perform MW monitoring in such operational contexts.

## 1. Introduction

Manned-Unmanned Teaming (MUM-T) can be seen as a cooperative teaming of multiple agents: several Unmanned Aerial Vehicles (UAVs) and possibly several manned aircrafts. The MUM-T organization described by Strenzke and collaborators contains multiple UAVs and a human operator present in a manned aircraft along with a flying pilot (Strenzke et al., 2011). However, our vision for the future of MUM-T missions is a team of several agents, in which an agent could be an artificial one—i.e., a UAV- or a human. Hence, in this context, the human agent is not considered as an operator controlling the UAV but rather as another team member participating equally as the other artificial agents. The implementation of such a MUM-T organization will require more cooperation and coordination between the agents, that could increase the mental workload of the human agent. However, there are immense advantages to this approach as for instance: benefiting from the faster and more calculative capabilities of the artificial agents, and for the human agents' better perception, judgment abilities and critical thinking (de Souza et al., 2020), increasing mission achievement chances while ensuring safety (Chanel et al., 2020b), or enabling a better proximity and state awareness of the human agents (Strenzke et al., 2011).

In human–multi-UAV interaction scenarios, interactivity is currently derived from the concept of autonomy (David and Nielsen, 2016). According to Goodrich and Schultz (2008), the simplest way to consider autonomy is by defining the level at which humans and robots interact and the scale to which each one of them is independent from the other. Therefore, the more autonomous and capable the artificial agents are to take decisions, the more they are capable of having a peer-to-peer interaction within a human-machine team. On the other hand, no autonomy means that a continuous presence of a human agent is required to drive a given machine.

There are several ways autonomy is defined and used, such as in the “adjustable autonomy” framework (Scerri and Reed, 2001; Luck et al., 2003; Bradshaw et al., 2004; Durand et al., 2009; Zieba et al., 2010) in which the division of labor between humans and artificial agents is not fixed but rather varying (Parasuraman, 2000). On the other hand, mixed-initiative systems have a dynamic autonomy allocation (Chanel et al., 2020c), in which the roles can or can not be determined in advance. Indeed, in this framework the roles and autonomy levels are derived with a flexible interaction strategy where each agent (human and artificial) can take control of the tasks they are best in Hearst et al. (1999). It can be seen as a teamwork-centered approach of adjustable autonomy. But, to fine-tune the interaction capabilities, the mixed-initiative approach can be seen as a hybrid of the teamwork-centered and predictive approach of adaptive autonomy. In which, the tasks are shared between both types of agents, and the authority to lead a task is negotiated based on the state (e.g., current capabilities) of each agent (Chanel et al., 2020b). The key idea of mixed-initiative systems is to let agents work more effectively, independently but cooperatively as a team.

### 1.1. Human Operator State

In order to design a mixed-initiative system, it is important to know what influences the human operator's (mental) state, including their decision making capabilities, and as a result, performance. Therefore, it is essential to know how to best analyze and take into account this mental state. There are several fundamental causes to every decision, sometimes it's more affected by either emotion (Angie et al., 2011), the level of intelligence (Leslau, 2010), the experience (Brockmann and Simmonds, 1997), or most important our evolution (Wilke and Todd, 2010). Most of the decisions one takes are usually unconsciously taken, even though one thinks of taking them with precise calculation and critical thinking (Soon et al., 2008). Within a human-machine interaction framework, the pendant to decision making in critical settings is human error and performance degradation in general. Human errors are one of the major factors of aviation mishaps—responsible for almost 60% of accidents depending on the system the human operator interacts with (Williams, 2004; Murphy, 2014). In this context, the errors are mostly linked to the automation design principles not being in accordance with human ergonomic principles (Dehais et al., 2015).

Human error is classically studied by means of behavioral and subjective measures. Yet, subjective and behavioral measures are known as overt measurements and are relatively limited as they do not allow for an uninterrupted/continuous and direct assessment of cognitive processes. However, physiological measures are a promising means to perform mental state monitoring in such a way (Mehta and Parasuraman, 2013). The recent field of neuroergonomics advocates for “*the study of brain and behaviour at work”* (Parasuraman, 2003), as it explores humans at work and in ecological settings through the lens of neuroscience. A promising venue would then be to exploit physiological metrics in addition to behavioral and subjective ones in order to improve our understanding of operators' mental state, through what is called *physiological computing* (Fairclough, 2008), but also to perform mental state monitoring (Roy et al., 2020). Indeed, these physiological metrics could be used as inputs to a system that would then adapt to the detected operator mental state. When based on cerebral activity only, these type of systems are called passive brain-computer interfaces (pBCIs). These systems are designed to estimate an operator's mental state (e.g., affective and/or cognitive state) based on their cerebral activity acquired through brain imaging methods such as electroencephalography (EEG). A given mental state is then estimated by applying machine learning methods onto the acquired brain activity features to adapt a system accordingly (George and Lécuyer, 2010; Zander and Jatzev, 2011). These systems have been successfully used to estimate a variety of cognitive and affective states such as attentional states, mental fatigue, and mental workload in laboratory settings (Brouwer et al., 2012; Roy et al., 2016a; Singh et al., 2018), but also, although less often, in close to or in real life settings (Borghini et al., 2014; Callan et al., 2015; Scholl et al., 2016; Verdière et al., 2018; Dehais et al., 2019a,b).

### 1.2. Mental Workload Assessment

Mental workload is defined as information processing capacity that is required to meet a system demand (Xie and Salvendy, 2000); or the difference of total information processing capacity and available information processing capacity at any given time (Gopher and Donchin, 1986); or it could be understood as task performance that leads to the reduction of capacity to perform another task requiring similar resources (Kramer et al., 1987). With regards to the previous section, mental workload could be considered as a medium of understanding the human operator's capabilities at a given point in time by a mixed-initiative system and use this knowledge to seize a given task if it favors mission safety or performance. The mental workload experienced by human pilots in MUM-T scenarios has already been thoroughly studied in the past, yet only through subjective metrics (Donath et al., 2010; Gangl et al., 2013a,b; Schulte et al., 2015) that do not allow for a continuous assessment, nor for a direct measure of the operator's cognitive state (Galdi et al., 2008; Plassmann et al., 2015; Bell et al., 2018).

Instantaneous Self Assessment (ISA) is a subjective mental workload assessment technique designed to get immediate subjective rating while performing a primary task (Tattersall and Foord, 1996). Whereas, behavior markers are the features obtained from the interaction of the participant and the system. They mostly comprises key strokes, response time, clicks, and performance scores (Chanel et al., 2020a). In which mental workload has an inverted U-shape effect on performance shown by Bruggen (Bruggen, 2015), and performance of working memory decline with higher difficulty level (Taylor et al., 2005; Gateau et al., 2015, 2018). Where as reaction time decreases with and increase in task difficulty (Sternberg, 1969; Gomarus et al., 2006). Moreover, a variety of physiological metrics can be used to evaluate an operator's mental workload, for instance derived from the following sensors (Heard et al., 2018): electroencephalography (EEG), electrocardiography (ECG),—that respectively record cerebral and cardiac activity, and eye-tracking (ET) that records oculo-motor behavior. Hence, regarding cerebral activity measures, the power modulations of different EEG frequency bands (e.g., *θ*: 4–8 Hz, *α*: 8–12 Hz, *β*: 13–30 Hz, and *γ*: 30–45 Hz) can be used for mental workload estimation (Roy et al., 2016a,b; Heard et al., 2018). Ratios of the power in these bands have been shown to be impacted by workload, such as the Engagement Index (EI) developed by Pope and collaborators (Pope et al., 1995; Berka et al., 2007; Chaouachi and Frasson, 2012) which is computed as follows: *EI* = *β*/(*α* + *θ*).

EEG's theta power tends to increase with increase in task demand, fatigue, focused attention, time pressure, sustained attention, and multi-tasking. It also increases with a decrease in vigilance (Borghini et al., 2014). Whereas, alpha power decreases with an increase in task demand, sustain attention, and multi-tasking. But it increases with an increase in fatigue and drowsiness, and a decrease in vigilance (Borghini et al., 2014). On the other hand, an increase in beta power is associated with the problem solving, judgement, and decision making capabilities of the human mind (Kumar and Bhuvaneswari, 2012). Beta power tends to increase with alertness and decrease with fatigue (Borghini et al., 2014). Likewise, increased gamma power is associated with a state of hyper alertness and integration of sensory inputs (Kumar and Bhuvaneswari, 2012).

As regards peripheral physiological measurements, ECG features in both the temporal and the frequency domains can be used for mental workload assessment. For instance, in the temporal domain, Heart Rate (HR) increases and Heart Rate Variability (HRV) decreases with an increase in mental workload (Heard et al., 2018). HRV can also be computed in the frequency domain however it cannot be computed on short windows and therefore is less useful for online mental state estimation in critical settings. Moreover, several ET features can also be used for mental workload estimation, such as blink frequency (number of blinks per minute) that is associated with cognitive and visual workload, blink latency (the amount of time between two blinks) that increases with mental workload, fixation duration (the amount of time the eyes fixated at a particular area) also increases with mental workload, and finally pupil dilation that also shows an increase with an increase in mental workload (Heard et al., 2018). Others sensor-related metrics have been suggested by Heard et al. (2018) that seem to be relevant to mental workload assessment. Interested readers can consult (Table 1 of Heard et al., 2018), for instance.

### 1.3. Physiological Signal Non-stationarity and Classification Methodology

Mental workload estimation using EEG has several advantages, although time has a notable effect on it. This phenomenon is mostly known as non-stationarity effect of EEG (Raza et al., 2019). In particular, the EEG signal even from same person can present different behavior with time. An instability in the raw wave form is noted by Shakhnarovich et al. (2007) in experiments spanning from hours to days. Alongside, large variations in signal amplitude was noted by Suner et al. (2005) within a day of recordings. Therefore, it eventually increases the difficulty in EEG-based mental workload classification as detailed by Roy et al. (2013, 2016b). In their studies, the accuracy using a Linear Discriminant Analysis on spectral features from a task-irrelevant 800-ms window decreased from 60 to 50% when training and testing datasets were separated with respect to time.

But in most BCI and pBCI research, the validation procedure of the classification pipelines tend to combine all the data and randomly select samples for training and for testing, irrespective of where they fall in the timeline of the experiment. As discussed earlier, time-on-task is one of the many factor of physiological signal non-stationarity. Therefore, this usual, or “traditional” classification validation procedure gives optimistic results with usually good offline mental workload estimation accuracy that usually exceeds 80% of accuracy on task-related windows (Brouwer et al., 2012; Dijksterhuis et al., 2013; Borghini et al., 2014). In these studies, the accumulated data from the whole experiment comprising all the conditions of a participant (in intra-subject classification) are divided into training and testing sets (Brouwer et al., 2012; Liu et al., 2017; Singh et al., 2018). However, in order to move towards the implementation of pBCIs in operational settings such as in MUM-T scenario, this factor has to be taken into account. Hence, the most ecological (i.e., close to real life implementation) way of creating and validating a classification pipeline best suited for real world scenarios, is to consider time and separate training and testing data based on time.

Based on this review of the literature, this study was designed to address two lacks and has the following objectives: (i) to characterize and estimate mental workload in a MUM-T setting with a search and rescue mission scenario; (ii) to assess the impact of the validation procedure on classification accuracy by comparing the results obtained for the traditional and the ecological methods; the ecological one was expected to yield lower accuracy.

In the next sections of this paper, the materials and methods section details the experimental protocol put in place in order to elicit two levels of mental workload within a MUM-T scenario, as well as the data acquisition and data processing steps. Then the results are presented, and lastly they are discussed along with recommendations and perspectives.

## 2. Materials and Methods

### 2.1. Participants

The experiment was performed on 14 healthy volunteers (6 females, 8 males; 24.4 y.o. ± 1.95). Before the experiment took place, all participants gave their written consent for data collection, storage and processing in an anonymous manner. The experiment was validated and authorized by the local ethical committee (CER Toulouse *Id number: 2019-137*).

### 2.2. Experimental Protocol

**Figure 1:**
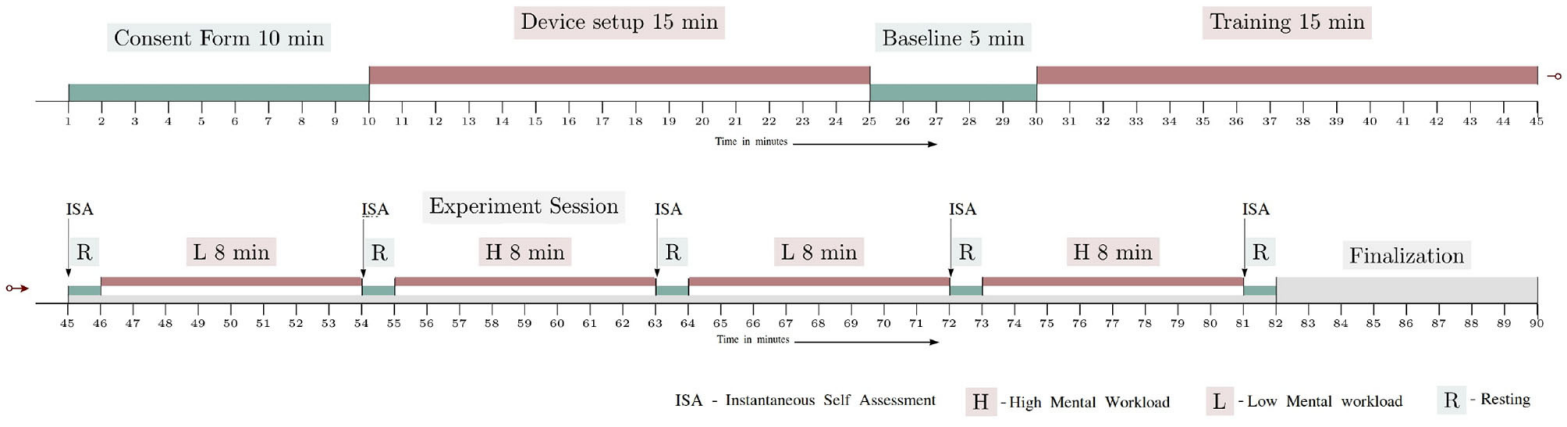
Experimental protocol timeline.

The experiment was divided into four blocks of 8 min each, each block corresponding to a S&R mission (i.e., there were four 8-min missions). Two blocks corresponded to the low mental workload condition (L), and the remaining two were for the high mental workload condition (H). Their order was pseudo-randomly changed between H-L-H-L and L-H-L-H for each participant. Each workload condition was separated by a 1 min break. Figure 1 illustrates the experimental protocol timeline. After having given their informed consent, and before starting the experiment, the participants were properly trained for the piloting task, and were also familiarized with a dummy session that included all the sub-tasks. This training was performed in order to familiarize the participants with the whole system and tasks to perform.

When they were comfortable with the simulator and the related sub-tasks, the instruction page was shown in the application screen to formally detail the experiment, its tasks, and the level of importance of each sub-task. Then, they were then told to press the start button whenever they were ready to start. In the very beginning of each break, the participants were asked to answer an interactive version of the Instantaneous Self Assessment (ISA) questionnaire (Tattersall and Foord, 1996) displayed in the application screen. The ISA questionnaire was shown with a slider that could be moved between 5 standard ISA options. Participants took 6 s in average to answer this questionnaire. After that, and for the remaining of the rest period, all four screens showed a black screen, except for screen 2 and 4 which showed a white dot on a black background. The participants were instructed to focus on the white dot in screen 4 during this phase. Therefore, the whole experiment lasted for 90 min.

### 2.3. Search and Rescue Mission

**Figure 2:**
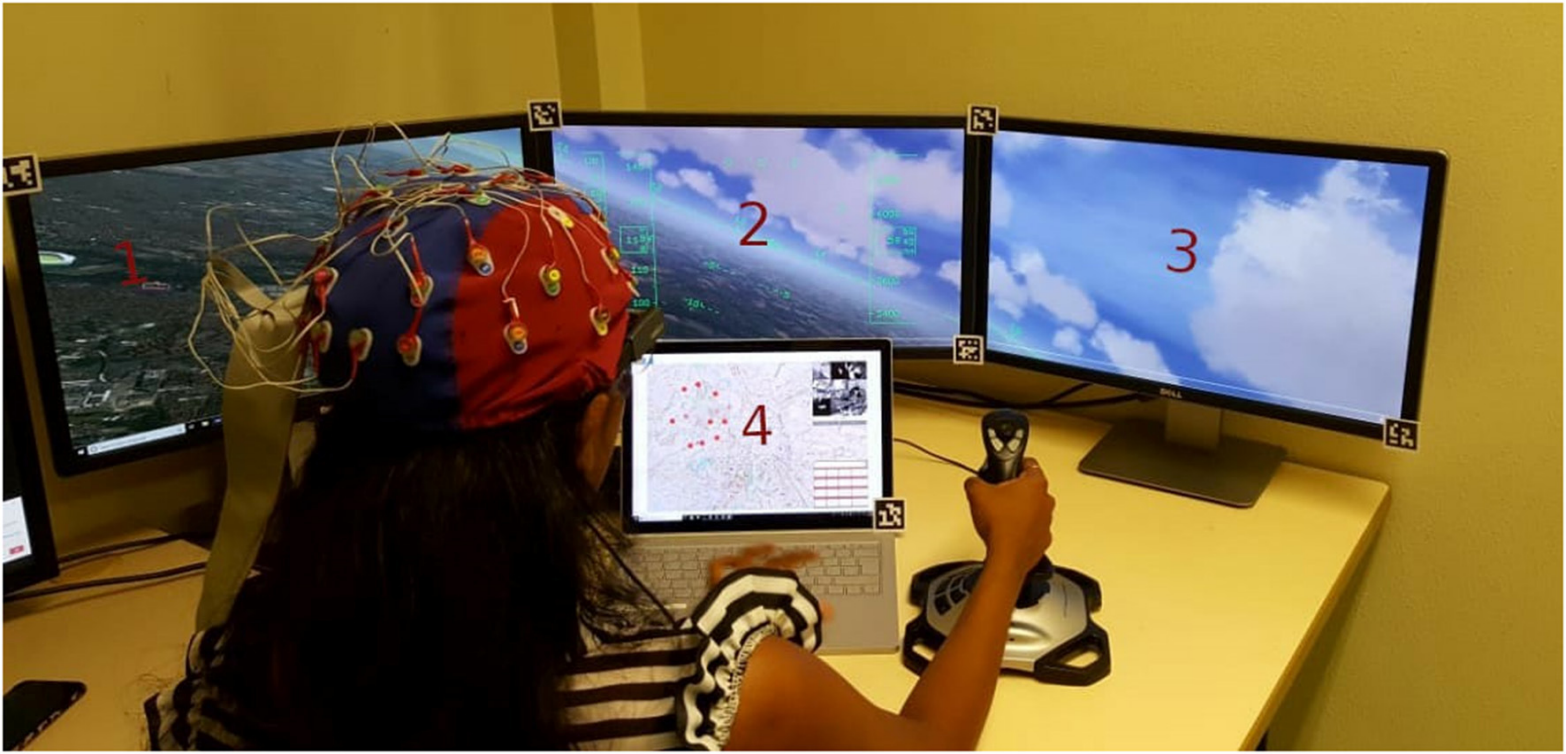
Aerofly simulatior and U-track application screens setup.

**Figure 3:**
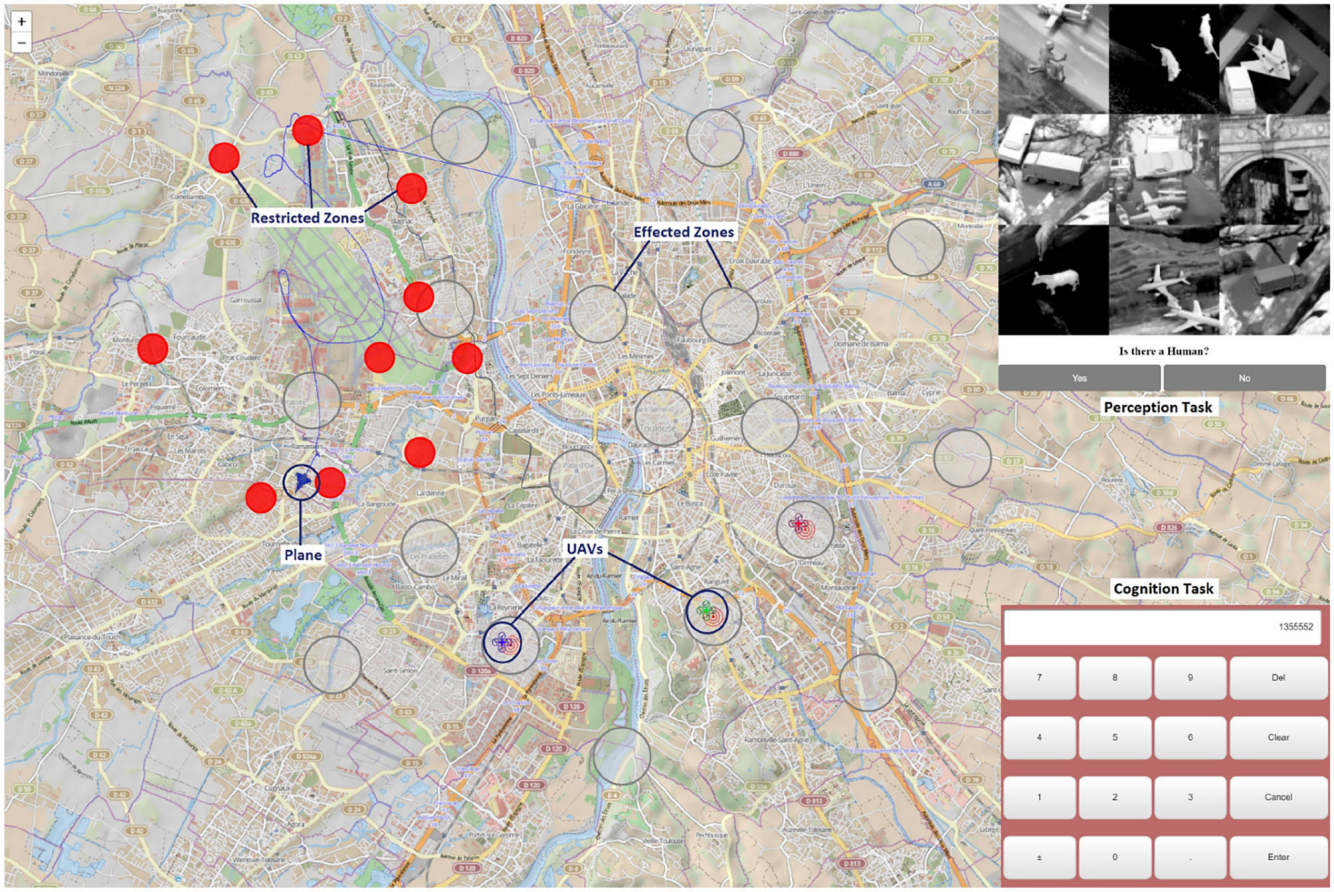
Zoom on the UAV application U-track used by the flying pilot to interact with UAVs.

**Figure 4:**
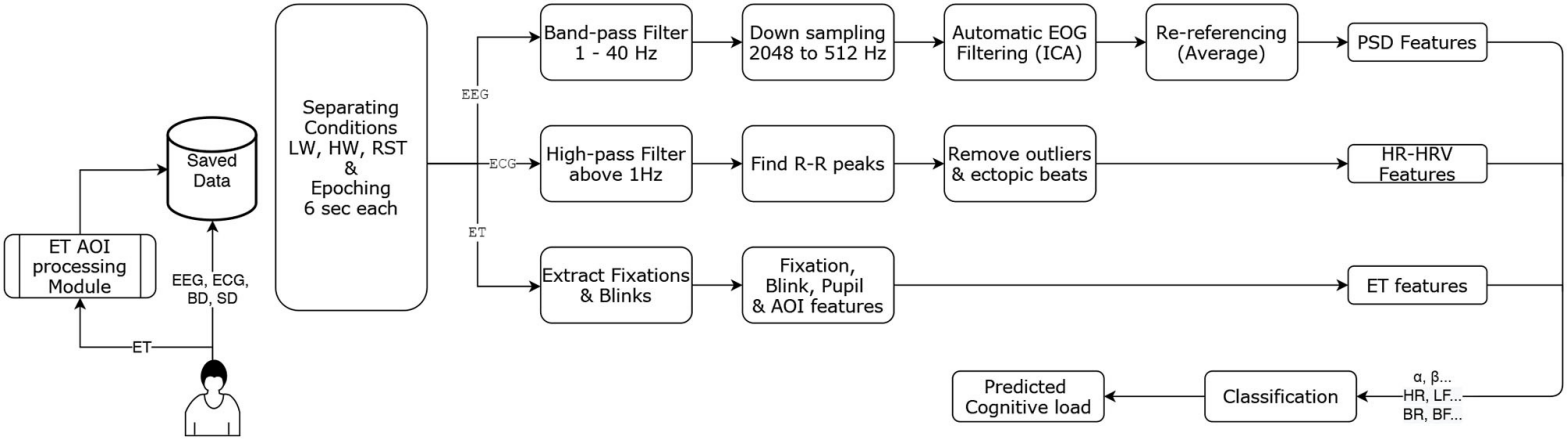
Pre-processing, feature extraction, and classification pipeline.

The task performed in this experiment was a search and rescue mission (S&R mission). During the mission, the participants had to perform a pilot flying task using a flight simulator (displayed in the first 3 screens in Figure 2) while interacting with the UAVs through the U-track application (see the 4th screen in Figure 2, or the Figure 3). The flight simulator that was used was the Aerofly FS2 flight simulator^1^. This flight simulator is commercially available, and possesses several functionalities such as: an advanced flight dynamics, a wide selection of aircraft, customizable flight conditions, highly detailed 3D cockpits, and options to add 3D structures in the environment. This functionality allowed us to create wrecked zones with destroyed 3D building inside the simulation. The main S&R mission task was composed of three sub-tasks: a detection and identification one, a working memory one, and a flying sub-task as detailed below:

- *Detection and identification sub-task:* simulated the process of UAV requests in the form of pop-ups in the U-track application (see Figure 2 screen 4, which corresponds to Area of Interest (AOI) 4.2 in Figure 4). A beep sound initiated the requests and the human pilot had to search if there was any human present in a group of 9 gray scale images (extracted from the Norb database LeCun et al., 2004), and had to answer with Yes or No. This was the highest priority sub-task, i.e., of priority level 1. The detection and identification request was an event-based sub-task with 15 identifications to be performed for each experimental condition. Note, the time interval between two requests was randomly chosen between 21 and 24 s.
- *Working memory sub-task:* consisted in Air Traffic Control (ATC) instructions played in the form of audio messages of headings to be followed by the pilot and communication channels for UAVs. In this task, the pilot was required to memorize communication channel values for the UAVs and to recall them in the U-track application using a numpad. This sub-task had the least priority, i.e., it was of priority level 3. Each ATC command was played with an time interval randomly chosen between 80 and 82 s. There were 6 ATC commands per experimental condition.
- *Pilot flying sub-task:* this sub-task was related to the continuous compliance with the ATC heading instructions. In other words, the human agent had to remember the ATC heading instructions and maintain that heading of the plane in the Aerofly simulator using the joystick while avoiding the restricted zones (in red in Figure 3). Maintaining the heading was of priority level 2 and avoiding the restricted zones was of priority level 1, equivalent to detection and identification sub-task's priority.

The mental workload level was varied by modifying the working memory and the flying sub-tasks. Indeed, the low mental workload (L) and high mental workload (H) conditions were different in: (i) the number of communication channel inputs (i.e., 1 vs. 2 randomly selected UAVs) and their value (i.e., the heading value plus 100 for one UAV communication channel in L vs. random values for the 2 UAVs communication channels in H), and, (ii) the placement of the restricted zones with respect to the plane's path (i.e., far from the path in L vs. close o the path in H). Additionally, the selected path (the sequence of ATC heading instructions) for the low mental workload condition comprised less sharper turns as compared to the high mental workload conditions.

### 2.4. Data Acquisition

Physiological and behavioral data from the participants were recorded thanks to three Lab Streaming Layer (LSL) streams that were created and published in a local network to be effectively recorded with LabRecorder, an LSL-based software^2^.

**Figure 5:**
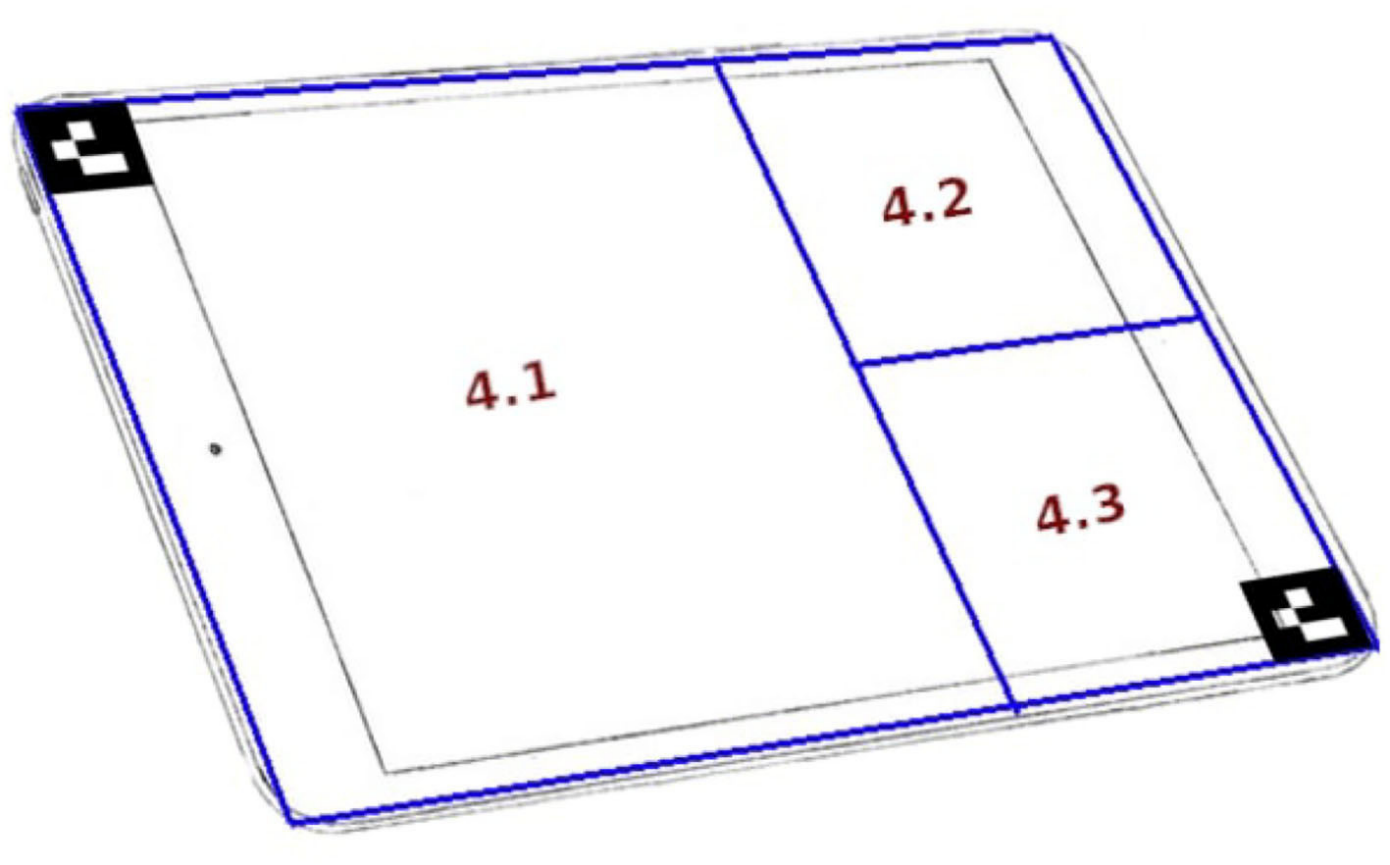
U-track application sub-areas of interest.

- The first LSL stream was devoted to electroencephalography (EEG), electro-oculography (EOG) and electrocardiogram (ECG) recordings. The EEG Biosemi system was used at 2,048 Hz, with 32 electrodes positioned following the standard 10/20 layout. Additionally, two electrodes were positioned below the right eye and at the outer canthi of the right eye to perform the EOG recordings. Lastly, two electrodes were placed on the participants' torso to perform the ECG recordings: 1 on the plexus and one on the left 5th intercostal.
- The second LSL stream was used to collect data from the Tobii glasses eye-tracker (ET). An application was developed to collect the raw data at 100 Hz and the image data at 25 Hz from the Tobii glasses using WiFi (Singh et al., 2020). This application synchronizes and processes, in real-time, image frames from image data and the raw gaze data to determine the gaze location with respect to specific Areas of Interest (AOIs), (see the “*ET AOI processing module”* shown in Figure 4). Note, this ET LSL stream contained almost 29 data fields, in which 27 were fed with Tobbi glasses raw data (e.g., timestamp, 2D gaze position, and pupil dilation of each eye), AOI number with gaze, and also the list of all Aruco codes visible in the image frame that help identifying AOIs. The image processing of AOIs using Aruco codes has been explained in detail in a previous work (Singh et al., 2020). Up to 7 AOIs were defined for this experiment, three AOIs for the Aerofly-related screens (one AOI per screen 1, 2, and 3 of Figure 2), three other AOIs concerning the U-track application (see Figure 5), and one AOI tagged as no-screen.
- The third LSL stream was devoted to the Aerofly simulator data, as well as the behavioral and subjective data grouped within the U-track application. This LSL stream was running at 100 Hz. It included all plane's parameters from the Aerofly simulator (e.g., speed, heading, and geolocation). The Aerofly data were sent to the U-track application and then embedded by the application in the same LSL along with other data fields. Note, the behavior and the subjective feedback data were event-based. It contained all the data regarding the human pilot interaction with the application, event time stamps, and response values for each sub-task. As event-based, they were only published in the LSL stream at their occurrence.

### 2.5. Pre-processing and Features Extraction

#### 2.5.1. Performance Scores

All three sub-tasks were scored. In the detection and identification pop-up sub-task, each correct detection was scored with 1. The pop-up response score and pop-up response time were calculated for each event and averaged per load condition, across all experimental blocks (i.e., yielding one value per load condition). The same procedure was applied for the working memory sub-task. However, the flying task score (*y*_*score*_) is based on the headings *x*_*n*_ observed every 500 ms. Thus, starting from an ATC command at time *t* until the next ATC command at time *t*+*k* the score is defined as the sum of Gaussian functions following the equation:

(1) $$\begin{array}{c} y_{score}=\sum_{n=t}^{t+k}\frac{1}{\sigma \sqrt{2\pi }}e^{-\frac{{\left(x_{n}-\mu \right)}^{2}}{2\sigma^{2}}}. \end{array}$$

Hence, participants scored higher when they flew in compliance with the required heading *μ*. The standard deviation *σ* was set at 20 degrees.

Alongside maintaining a correct heading, there was a higher priority part of the flying task, that was to avoid the restricted zones. Thus, for each 50 ms in the restricted zone, a penalty of 0.5 was applied to the heading score. Note that the score of the flying sub-task was a continuous score computed from the ending of an ATC command call until the next ATC call. At each new ATC call, the score related to the previous ATC command was published in the LSL stream. The scores related to all ATC calls in a given workload condition were then averaged, giving one score value per load condition.

#### 2.5.2. Subjective Feedback

The subjective feedback measure was based on the results of the Instantaneous Self-assessment (ISA) (Tattersall and Foord, 1996) questionnaire launched at the end of each mission (i.e., experimental block). The obtained score ranged from 0—Under Utilized—to 100—Excessive. The ISA scores were averaged per mental workload condition.

#### 2.5.3. Physiological Data Processing

Figure 4 illustrates the offline physiological data processing flow. On the very first step, participant's data were separated for each load or rest condition. As seen earlier, there were two low mental workload and two high mental workload conditions of 8 min each; and 5 resting conditions of 1 min each. Therefore, 9 blocks of conditions for each participant were created. Then, each block was processed one by one to create 6-s epochs. An important thing to note is, EEG and ECG were coming from same stream (Biosemi stream), but the ET data was coming from a different stream. Therefore, by using timestamps from the Biosemi stream, which was of highest frequency (2,048 Hz), timestamps for the beginning and the ending of the 6-s epoch were selected as reference timestamp. These selected timestamps were then used to get closest timestamps from the ET stream. Each epoch was then fed to their respective sub-pipelined for further processing, as detailed below:

- EEG epochs were processed using python's MNE library ^3^. Each 6-s epoch was first passed through a band-pass filter for filtering out frequencies below 1 Hz and above 40 Hz, and then downsampled to 512 Hz. Ocular artifacts were automatically removed based on an Independent Component Analysis (ICA, fastica algorithm) using EOG channels as reference signals. Then, an average re-referencing was applied to the cleaned data. Cleaned epochs were then used to extract PSD features using the multitaper method. The extracted features are the absolute power in the following bands: *θ* (4–8 Hz), *α* (8–12 Hz), *β* (12–30 Hz), and *γ* (30–45 Hz).
- ECG 6-s epochs were passed through a high-pass filter (above 1 Hz), then peak detection was performed and R-R intervals were extracted. The Aura-healthcare^4^ Python package was used to remove RR-interval outliers using the Malik rule (Kamath and Fallen, 1995), and then finally heart-rate (HR) and heart-rate variability (HRV) temporal features were extracted using the same package. In particular, HRV is approximated using the Standard Deviation of the Normal-to-Normal (SDNN) RR-intervals. These HR and HRV temporal features were normalized with respect to the previous resting period by subtracting each feature with the average value of this feature in the resting condition.
- ET 6-s epochs were first processed to extract fixations and blinks. Using Tobii glasses, the data was sampled at 100 Hz and the samples that were missing from continuous data stream were inferred as blinks. Fixations are generally considered as the windows between two consecutive saccades (i.e., rapid eye movements between 2 points) (Nyström and Holmqvist, 2010); here these fixations were extracted using the pygaze python library (Dalmaijer et al., 2014).

### 2.6. Classification

A feature selection approach based on the statistical analyses was used to focus on the most promising features for performing the classification step. Hence, only features for which there was a significant effect of the load condition were kept for the classification stage (see section 2.7). Each feature vector (sample) considered for classification was extracted from non-overlapping 6-s epochs. To evaluate the impact of features grouped by the sensor used to acquire them, 7 combinations of features were considered: EEG-only features, ECG-only features, ET-only features, EEG and ECG features, EEG and ET features, ECG and ET features, and finally EEG, ECG, and ET features. Moreover, due to technical issues during the acquisition for two participants, the EEG and ECG data of only 14 participants were kept for the analysis, and 13 for the ET data.

Well-known classifiers that can be applied on small datasets were used: k-Nearest Neighbors (kNN), Decision Trees (DT), AdaBoost (AB), Gaussian Naive Bayes (GNB), Linear Discriminant Analyses (LDA), Quadratic Discriminant Analysis (QDA), and Support Vector Machine (SVM). Hence, we chose not to employ highly data demanding classification algorithms like deep learning, but also did not use Riemannian methods that—to our knowledge—are seldom used with recording methods other than EEG. Indeed the main focus here was to perform a multi-modal estimation. The training-test designs and cross-validation methodologies—traditional vs. ecological—that were compared are explained in detail in the following.

#### 2.6.1. Traditional Classification Design

In this setting, the data was classified in a traditional manner, that is to say with all the data is pooled together, shuffled and splitted into training data and testing data sets.

**Intra-subject:** For intra-subject classification in the traditional design, 70 percent of samples were selected from each participant's data and used for parameters optimization using a grid search method with 5 cross-validations. Then, the best parameters were selected and used with all the data in a 10-fold cross-validation method.

**Inter-subject:** For inter-subject classification in the traditional design, a *leave-n-out* cross-validation was used, with *n* = 2. Hence, 2 participants were selected each time for testing, and the remaining 11 participants were used for training. Therefore, every time the grid search method was used on selected training data of 11 participants for parameter optimization with 5 cross-validation. These selected parameters were used to train the model with training data and tested on the 2 selected participants for testing separately.

#### 2.6.2. Ecological Classification Design

To take into account the non-stationarity effects of EEG signals linked to time, a more ecological and realistic form of classification evaluation was designed by separating training and testing data with respect to time, as explained in the following.

**Figure 6:**
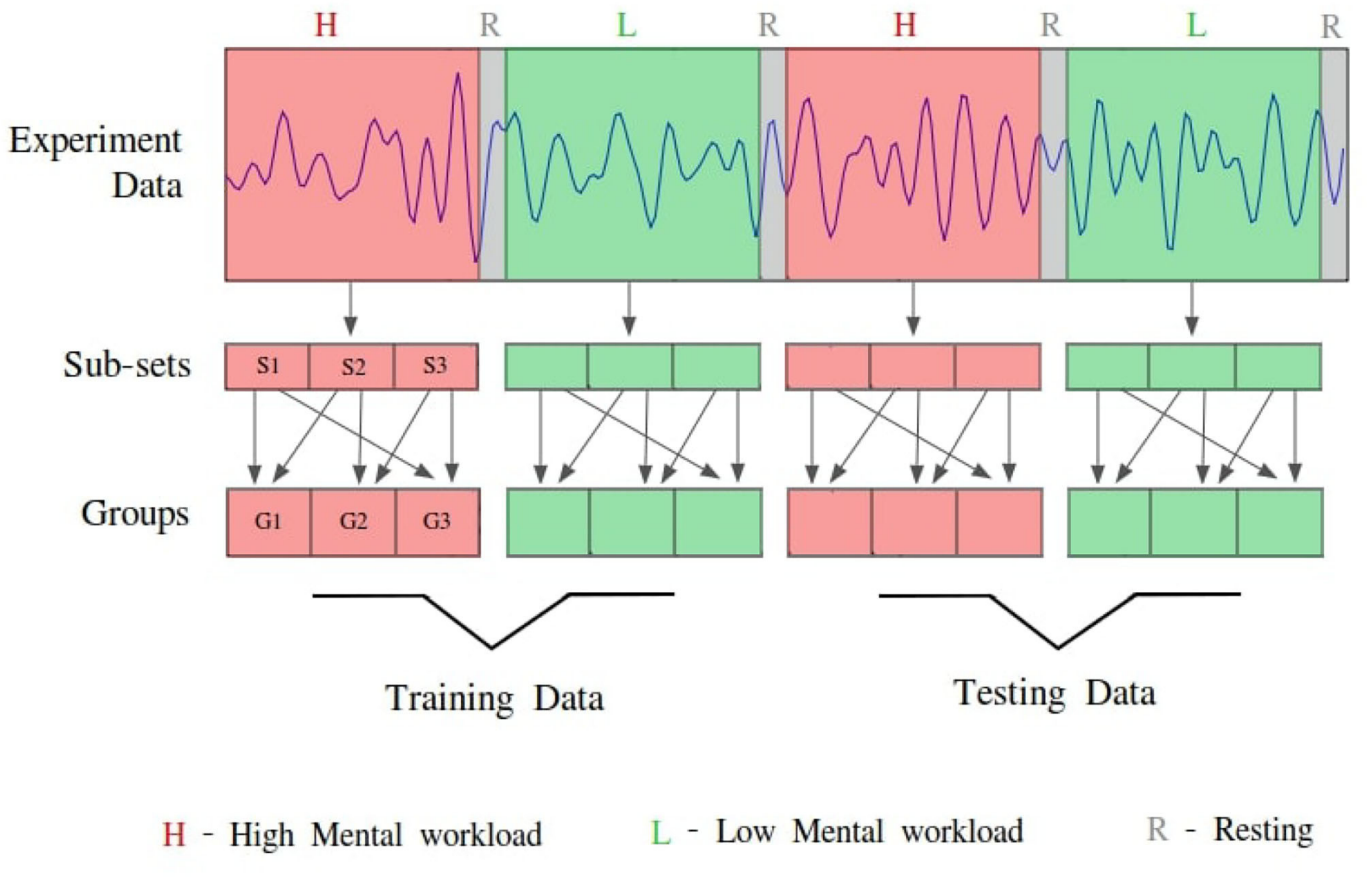
Allocation process of each participant's data in the ecological classification validation design for creating testing and training sets. Data from each condition (High:H, Low:L) are divided into three subsets (S1, S2, and S3). These subsets are used to create three groups (G1, G2, and G3; e.g., G1 = S1 + S2).

**Intra-subject:** For intra-subject classification in the ecological design, the initial two blocks of mental workload data (one of Low and one of High) were used for training and the remaining two conditions blocks were used for testing the classifiers' accuracy. To ensure a total separation of training and testing sets, a custom made cross-validation was designed to meet the needs of this ecological design. In this validation methodology, each mental workload condition (Low and High) from the training and testing sets were divided into 3 smaller sub-sets. Then using these sub-sets three groups were created. Please refer to Figure 6 illustrating the sub-sets and groups creation for this cross-validation method. For instance, the group *G*1 contained sub-set *S*1 and *S*2, group *G*2 contained sub-set *S*2 and *S*3, and group *G*3 contained sub-set *S*3 and *S*1, for each load condition from both training and testing sets. Then two groups were pseudo-randomly selected from the training set and used for parameters optimization (grid search) with 5 cross-validations and following it, the classifier was trained. Then the test phase was performed with all 9 possible combinations of test groups (i.e., taking 1 from Low and 1 from High load conditions it is possible to create 9 combinations). This training and testing procedure was repeated with all the 9 combinations of training set, which eventually gave 81 validation scores for each classifier.

**Inter-subject:** For inter-subject classification in ecological design, a particular *leave-n-out* cross validation was used, with *n* = 2. In this procedure, each time only the last 2 blocks of mental workload data (one Low and one High) of 2 participants were selected for testing. The remaining 11 participants along with the initial 2 blocks of mental workload data of 2 testing participants were used for training. Therefore, the combination of training data is used for parameters optimization (grid search) with 5 cross-validations, and following the classifier was trained, and finally tested with the 2 selected participants separately.

### 2.7. Statistical Analysis

Statistical analyses were performed on the subjective, behavioral, physiological and classification data using the Statistica software, in order to assess the impact of the experimental conditions and used features and techniques. Paired *t*-tests and Wilcoxon tests were used for subjective and behavioral data analysis, and for ECG features depending on the respect or not of the data distribution normality assumption.

Regarding the EEG data analysis, in order to decrease the dimension of the dataset, 20 electrodes that had a highest magnitude difference between high and low mental workload conditions were kept: Fp1, AF3, F7, F3, FC5, T7, CP5, P7, PO3, O1, Oz, O2, PO4, P8, T8, FC6, F4, F8, AF4, and Fp2. A repeated measure n-way ANOVA was applied on the EEG power features (i.e., 2 load conditions × 20 electrodes × 4 frequency bands—*θ*, *α*, *β*, and *γ*). The sphericity assumption was checked and a Greenhouse-Geiser correction was applied when violated. Tukey *post-hoc* tests were performed for each statistically significant main effect and interaction effect.

Concerning the ET features, two different analyses were performed. A first one was performed on features that are assumed to be unrealted, i.e., blink latency, the number of fixations, the fixation duration, and pupil dilation. For these, paired *t*-tests or Wilcoxon tests were used based on the normality of the data. The other features that have within relation, such as the number of fixations on each AOI and the total fixation duration on each AOI were analyzed with a repeated n-way ANOVA. The main objective of this analysis is to identify if there exists any statistical difference between those ET features given the two mental workload conditions, and AOI (e.g., 2 load conditions × 5 areas of interest). Note that there are 7 AOIs in total along with one “no screen” AOI. However, two AOIs were not used because they contained almost no fixations.

Lastly, an ANOVA was performed on the classification results to assess the impact of the type of validation pipeline -either traditional or ecological- on the classification accuracy reached using the various features and classifiers for both the intra-participant and the inter-participant estimations (i.e., 2 validation pipelines × 7 classifiers × 7 feature combinations). Tukey *post-hoc* tests were performed for each statistically significant main effect and interaction effect. Additionally, the classification results were compared with the chance level adjusted with respect to the amount of data (Müller-Putz et al., 2008).

## 3. Results

### 3.1. Subjective and Behavioral Data

**Figure 7:**
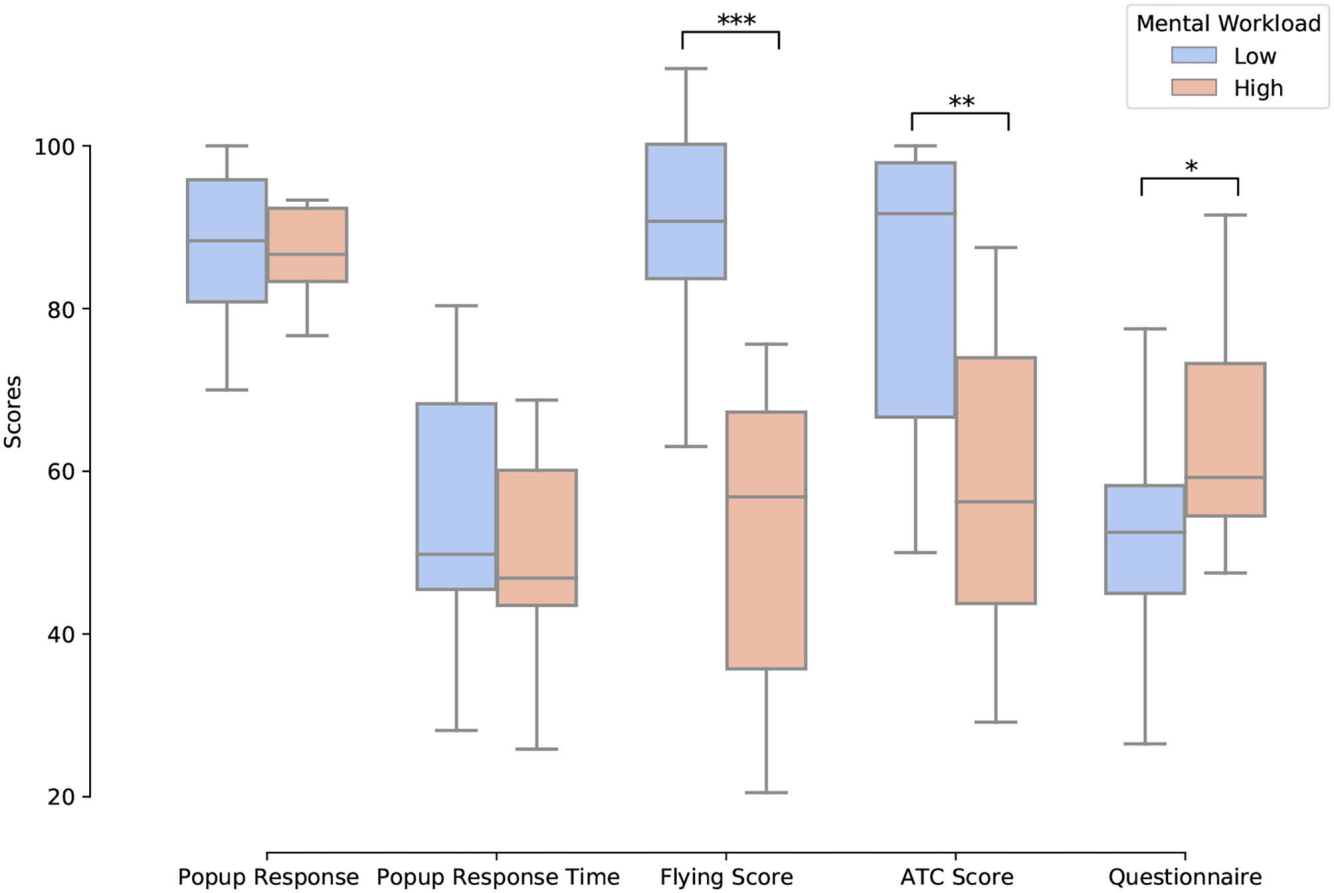
Illustration of the statistical results for the subjective and behavioral metrics for 14 participants using Paired *t*-test. ***p < 0.001, **p < 0.01, and *p < 0.05. Pop-up response time is multiplied by 10 for visualization purposes.

The analysis of the subjective and behavioral data showed that performance decreased, reaction times increased, and the reported workload increased with an increase in mental workload (i.e., task difficulty; see Figure 7). Indeed, the reported workload acquired through the ISA questionnaire significantly increased with load (*t* = 3.26, *p* < 0.05). Moreover, the pilot flying and ATC commands related scores significantly decrease with load (*t* = −4.77, *p* < 0.001 and *t* = −3.79, *p* < 0.01, respectively).

### 3.2. Physiological Data

#### 3.2.1. Electrocardiogram (ECG)

**Figure 8:**
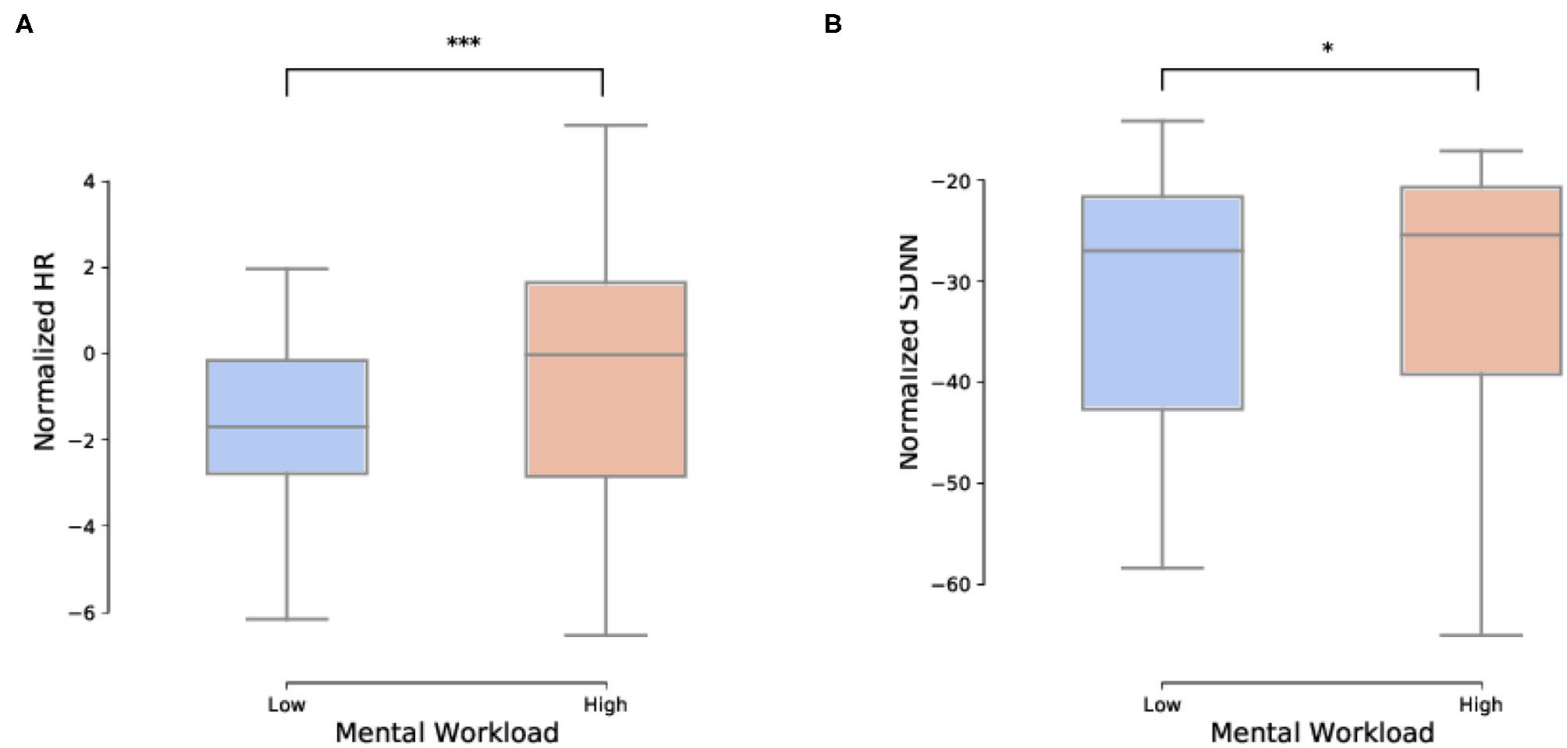
Impact of load on the ECG temporal features. **(A)** Impact of load on the normalized heart rate (HR) temporal feature (*p* < 0.001). **(B)** Impact of load on the normalized heart rate variability (HRV) (*p* < 0.05). Specifically, HRV is the Standard Deviation of the Normal-to-Normal (SDNN) RR-interval. ***p < 0.001 and *p < 0.05.

A paired *t*−test for Heart Rate data and a paired Wilcoxon test for Heart Rate Variability were performed with respect to the two conditions (low and high mental workload, see Figures 8A,B). There was a significant effect of load on both metrics with a significant increase in HR (*t* = 27, *p* < 0.001) and a significant decrease in HRV (*W* = 0.28, *p* < 0.05) when load increased.

#### 3.2.2. Eye-Tracking (ET)

The impact of the load condition on the eye-tracking features extracted using the processing pipeline (blink latency, number of fixations, fixation duration, and pupil dilation) was statistically assessed using paired *t*-tests. Aside from blink latency, all the other features were significantly impacted by load. Hence, with an increase in load, the number of fixations significantly increased (*t* = 3.15, *p* < 0.01), the average fixation duration significantly decreased (*t* = 2.79, *p* < 0.05), and pupil dilation significantly increased (*t* = 3.83, *p* < 0.01).

**Figure 9:**
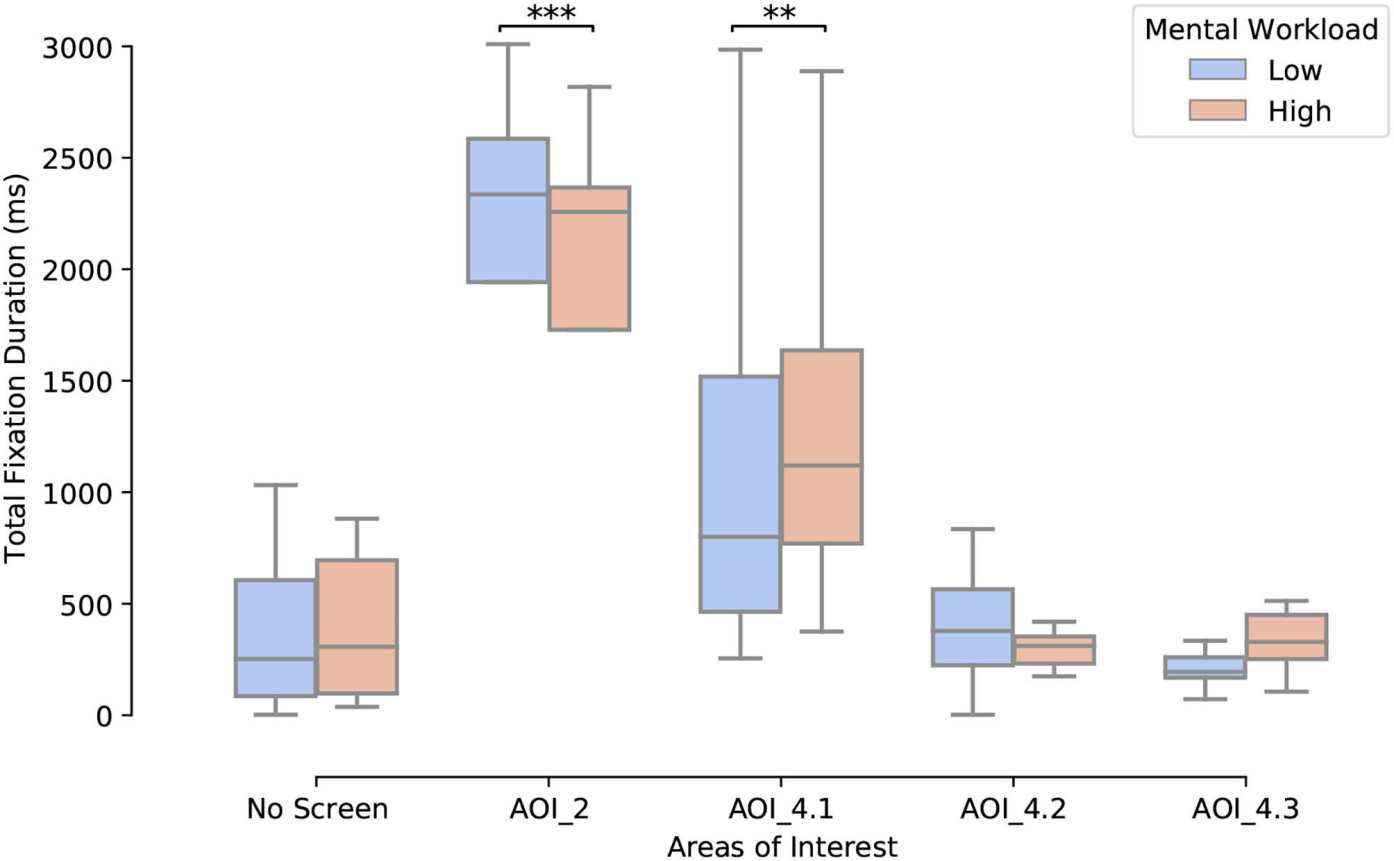
Impact of the mental workload condition on the total duration of fixations per AOI (**p < 0.01, ***p < 0.001).

The impact of load on the number of fixations and the total fixation duration features for each AOI was also statistically assessed using an n-way ANOVA. There was a significant main effect of load [*F*_(1,12)_ = 5.39, *p* < 0.05], and of AOIs [*F*_(4, 48)_ = 15.13, *p* < 0.001], as well as a significant interaction effect [*F*_(4, 48)_ = 12.08, *p* < 0.001] on the total fixation duration (Figure 9). Hence, in the high load condition participants spent more time fixating AOI 2 (middle screen of the plane flying simulator) and AOI 4.1 (U-track sub-part for analyzing the movement of plane to avoid red zones) than in the low load condition (*p* < 0.01).

Regarding the number of fixations, there was also a significant main effect of load [*F*_(1,12)_ = 9.97, *p* < 0.01], of AOI [*F*_(4, 48)_ = 11.87, *p* < 0.001] and a significant interaction effect [*F*_(4, 48)_ = 6.79, *p* < 0.001]. Hence, participants made more fixations on the AOI 4.1 in the high workload condition than in the low workload one (*mean*_*low*_ = 2.28; *std*_*low*_ = 1.70; *mean*_*high*_ = 2.72; *std*_*high*_ = 1.63; *p* < 0.01).

#### 3.2.3. Electroencephalography (EEG)

**Figure 10:**
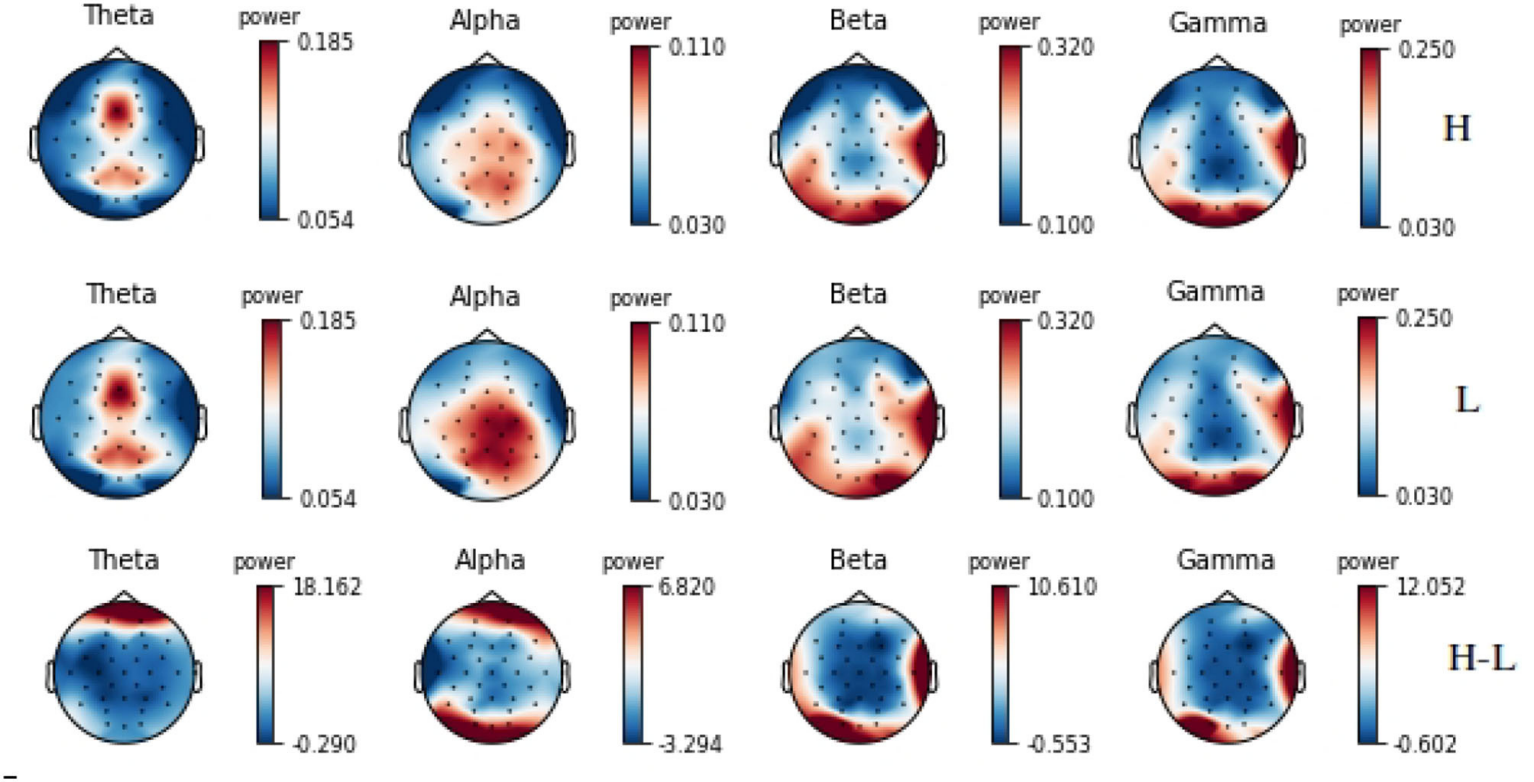
Topographic maps of the average power across subjects in the *θ*, *α*, *β*, and *γ* bands for the high and low mental workload conditions, and their difference (“H”: high workload; “L”: low workload; “H-L”: difference between high and low workload).

Mental workload had a significant impact on the EEG features (i.e., the power in the 4 frequency bands of interest), with all ANOVA factors and interactions being significant. In particular, there was a significant main effect of load [*F*_(1,13)_ = 16.31, *p* < 0.01] with a general increase in power with an increase in workload. However this effect was modulated by interactions with electrode site [*F*_(19,247)_ = 1.98, *p* < 0.001], band [*F*_(3,39)_ = 16.17, *p* < 0.001], and electrode site and band [*F*_(57,741)_ = 2.45, *p* < 0.001]. Indeed, only the power of the beta and gamma bands did significantly increase with an increase in load at the following 10 electrode sites: Fp2, FC5, FC6, T7, T8, CP5, P8, O1, Oz, and O2 (*p* < 0.01), that is to say mostly at fronto-central, temporal, and occipital sites (Figure 10 for illustration). The decrease in alpha power with an increase in load observed at parietal sites did not reach significance.

Moreover, the Engagement Index (EI) significantly increased with an increase in workload [*F*_(1,13)_ = 9.65, *p* < 0.01], with a significant interaction between load and electrodes [*F*_(19,247)_ = 3.37, *p* < 0.01]. Hence, the EI significantly increased with load at the FC5, FC6, T7, T8, CP5, and P8 electrode sites (*p* < 0.05).

### 3.3. Classification Results

**Figure 11:**
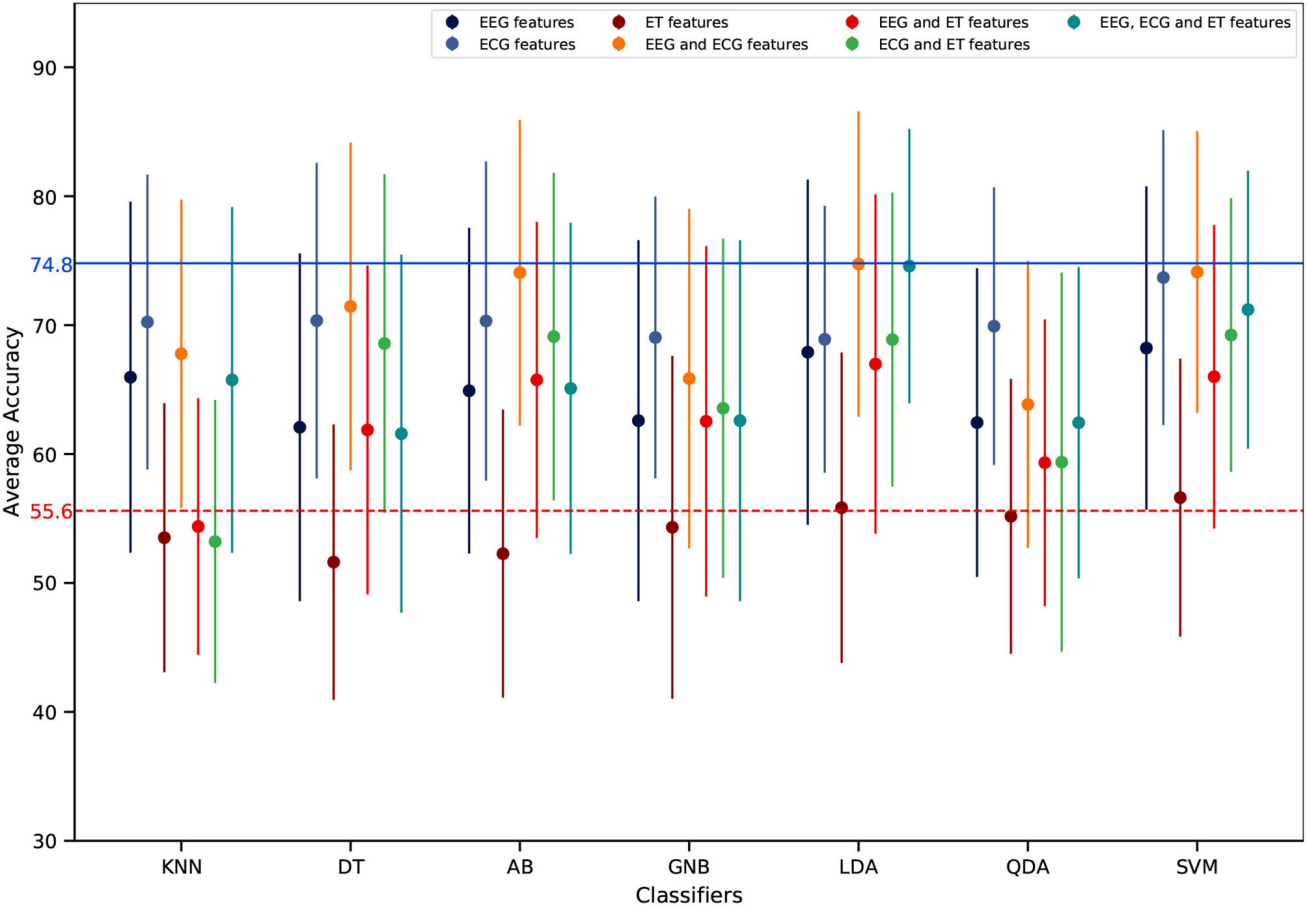
Intra-subject mental workload estimation results per features and classifiers with the traditional validation design (average across participants). The dots represent the average score of each feature combination and the vertical line represents the associated standard deviation. The dashed horizontal red line represents the adjusted chance level while the blue one represents the highest classification accuracy.

**Figure 12:**
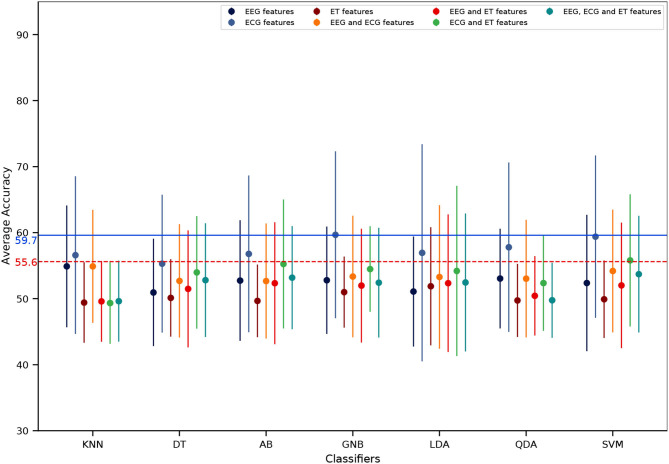
Intra-subject mental workload estimation results per features and classifiers with the ecological validation design (average across participants). The dots represent the average score of each feature combination and the vertical line represents the associated standard deviation. The dashed horizontal red line represents the adjusted chance level while the blue one represents the highest classification accuracy.

**Figure 13:**
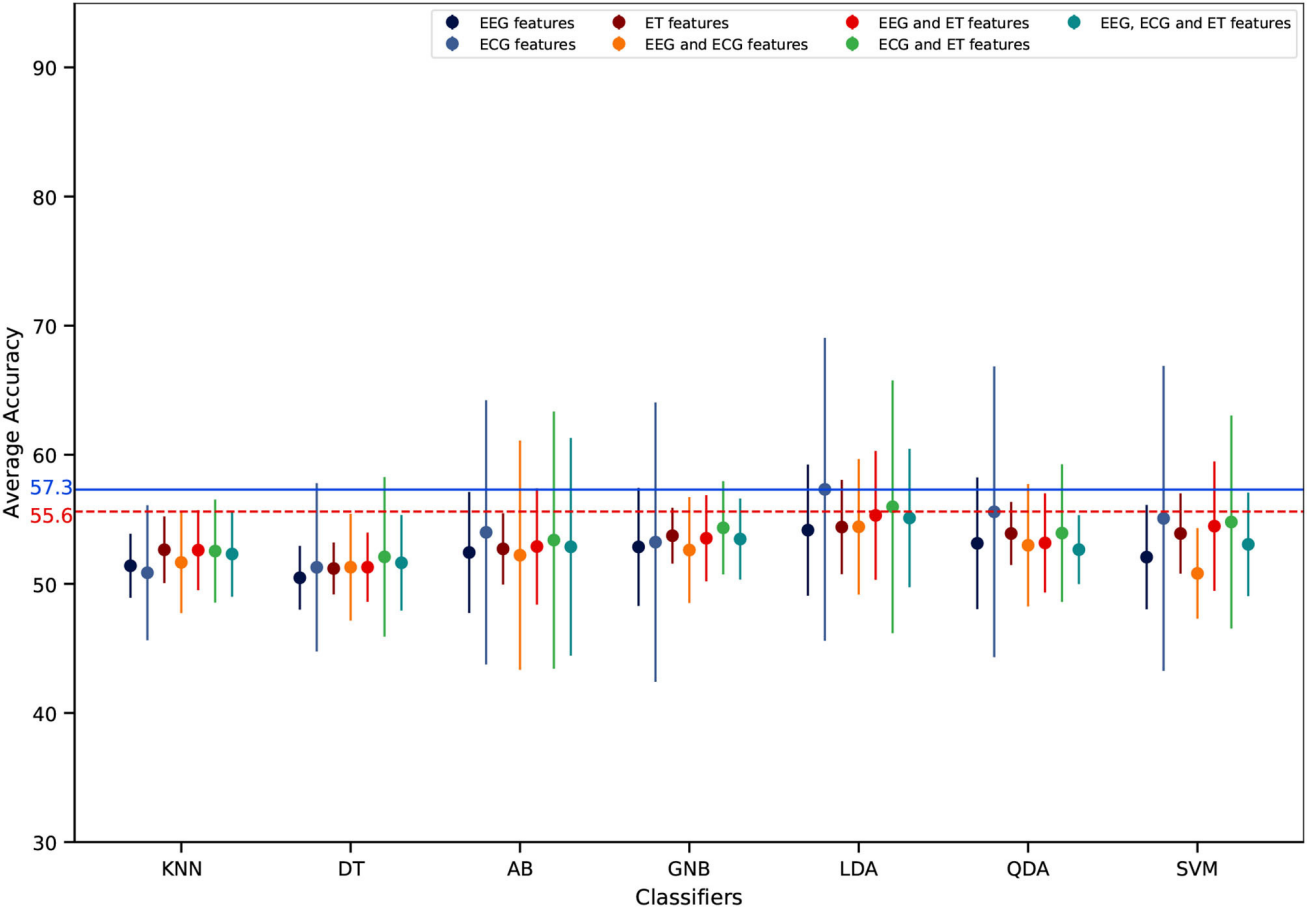
Inter-subject mental workload estimation results per features and classifiers with the traditional validation design (average across participants). The dots represent the average score of each feature combination and the vertical line represents the associated standard deviation. The dashed horizontal red line represents the adjusted chance level while the blue one represents the highest classification accuracy.

**Figure 14:**
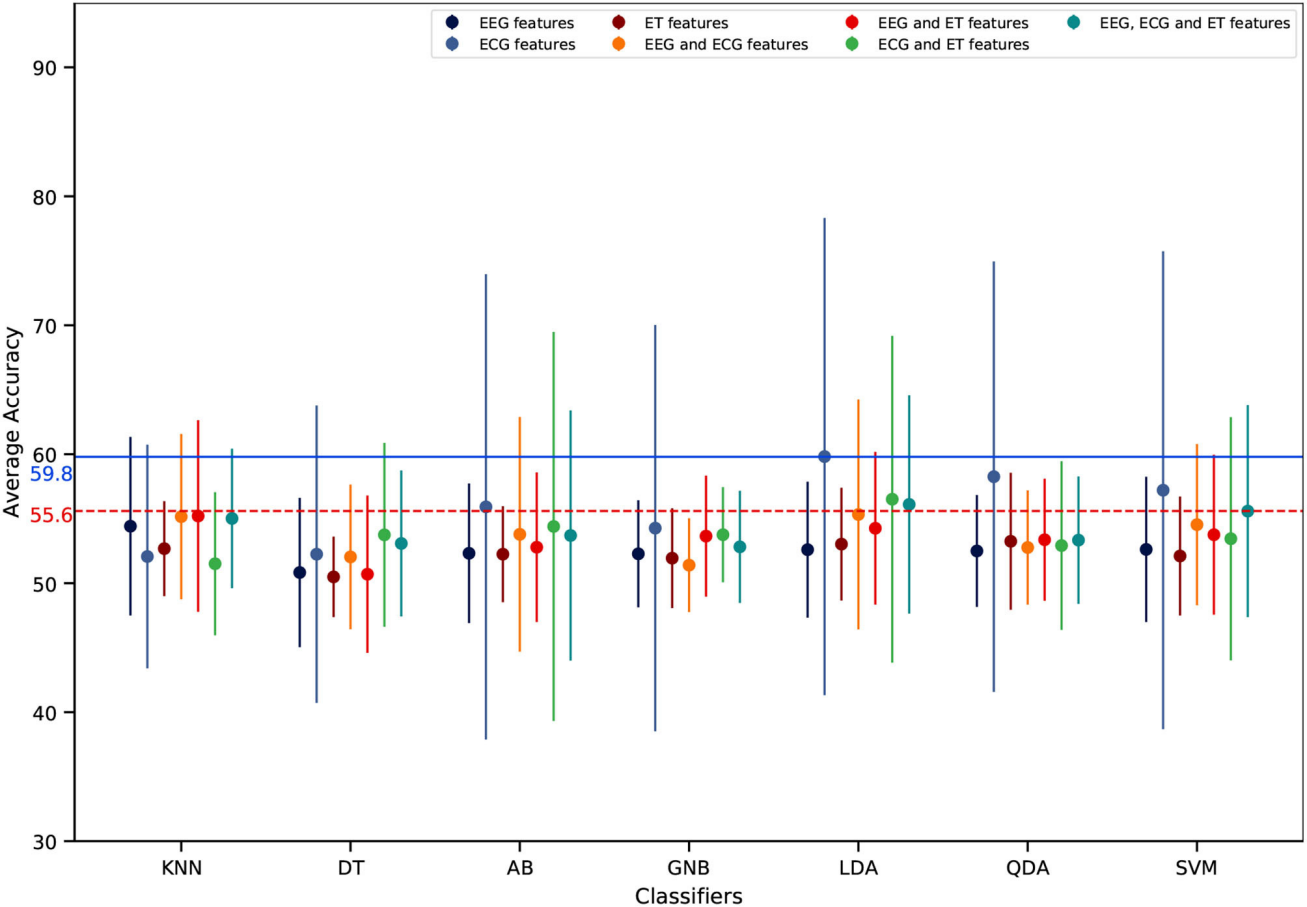
Inter-subject mental workload estimation results per features and classifiers with the ecological validation design (average across participants). The dots represent the average score of each feature combination and the vertical line represents the associated standard deviation. The dashed horizontal red line represents the adjusted chance level while the blue one represents the highest classification accuracy.

Regarding mental workload estimation results, the classification accuracy was obtained for four different validation methods: the traditional intra-subject classification design (see Figure 11), the ecological intra-subject classification design (see Figure 12), the traditional inter-subject classification design (see Figure 13), and the ecological inter-subject classification design (see Figure 14). The results are detailed in these figures which also include an adjusted chance level that takes into account the number of trials per class (Müller-Putz et al., 2008). A detailed comparison for all features, classifiers, and validation design combinations is also given in Table 1. From these results and from the n-way repeated measures ANOVAs performed to assess the impact of the validation method, the features used for classification and the classifier, the following can be said:

- **Intra-subject classification**: the highest accuracy was achieved with the traditional validation design with 74.8% (Figure 11) as compared to 59.6% with the ecological validation design (Figure 12). With the traditional validation design all features and classifiers gave estimation results above the adjusted chance level, except using ET-only features for all classifiers, and EEG and ET as well as ECG and ET with the KNN classifier. With the ecological validation design, only a few features and classifiers gave results above the adjusted chance level, i.e., ECG-only features for all classifiers except DT. There was a significant drop in classification accuracy from the traditional validation design to the ecological one [*F*_(1, 12)_ = 100.92, *p* < 0.001]. Also, there were significant main effects of the features used [*F*_(6, 72)_ = 9.91, *p* < 0.001], the classifier used [*F*_(6, 72)_ = 11.18, *p* < 0.001], as well as significant interactions between the validation method and the features [*F*_(6, 72)_ = 4.95, *p* < 0.001), the validation method and the classifier [*F*_(6, 72)_ = 11.85, *p* < 0.001], the features and the classifier [*F*_(36, 432)_ = 5.74, *p* < 0.001], as well as the double interaction between the validation method, the features and the classifier [*F*_(36, 432)_ = 3.62, *p* < 0.001]. Indeed, whatever the classifier and the validation design the ECG-only features gave significantly higher estimation accuracy, and ET-only the worst (*p* < 0.05). However the EEG and ECG feature sets also allowed to reach high classification accuracy with the traditional design with AB, LDA, and SVM (*p* < 0.05), and all three feature types combined with the LDA (*p* < 0.05). Indeed, regarding the classifiers, best performance was reached with the traditional validation design using the AB, LDA, and SVM ones, LDA and SVM reaching maximum performance (*p* < 0.05); no difference between classifiers was significant with the ecological validation design.
- **Inter-subject classification**: the highest accuracy was achieved with the ecological validation design with 59.8% (Figure 14) as compared to 57.4% with the traditional design (Figure 13). Interestingly, 59.8% of accuracy is also higher than the intra-subject classification accuracy achieved in ecological settings. There was no significant impact of the validation method, nor the features used. There was only a main effect of the classifier used [*F*_(6,72)_ = 2.73, *p* < 0.05], with LDA giving significantly higher accuracy than DT (*p* < 0.01). Although there was no significant effect of the features used, with the traditional validation design the best performance was reached using ECG-only features which was the only feature set giving results above the adjusted chance level. Moreover, with the ecological validation design ECG features were also giving the best estimation performance with accuracy above chance level reached only with this feature set for LDA, QDA, and SVM, with the exception of EEG and ECG, and all three feature types with the LDA that also gave results above the adjusted chance level.

**Table 1:** Comparison of different features and classifiers for traditional and ecological validation designs (mean accuracy and standard deviation).

			**EEG**	**ECG**	**ET**	**EEG**	**EEG**	**ECG**	**EEG,**
						**and ECG**	**and ET**	**and ET**	**ECG, and ET**
Traditional	Intra	KNN	66.0 ± 13.6	70.2 ± 11.4	53.5 ± 10.4	67.8 ± 12.0	54.4 ± 10.0	53.2 ± 11.0	65.7 ± 13.4
		DT	62.1 ± 13.5	70.4 ± 12.2	51.6 ± 10.7	71.4 ± 12.7	61.9 ± 12.8	68.6 ± 13.1	61.6 ± 13.9
		AB	64.9 ± 12.6	70.3 ± 12.4	52.3 ± 11.2	74.1 ± 11.9	65.7 ± 12.3	69.1 ± 12.7	65.1 ± 12.8
		GNB	62.6 ± 14.0	69.0 ± 10.9	54.3 ± 13.3	65.9 ± 13.2	62.5 ± 13.6	63.6 ± 13.2	62.6 ± 14.0
		LDA	67.9 ± 13.4	68.9 ± 10.4	55.8 ± 12.0	**74.8** **±** **11.8**	**67.0** **±** **13.2**	68.9 ± 11.4	**74.6** **±** **10.6**
		QDA	62.4 ± 12.0	69.9 ± 10.8	55.2 ± 10.7	63.8 ± 11.1	59.3 ± 11.1	59.4 ± 14.7	62.4 ± 12.1
		SVM	**68.2** **±** **12.5**	**73.7** **±** **11.4**	**56.6** **±** **10.8**	74.1 ± 10.9	66.0 ± 11.8	**69.2** **±** **10.6**	71.2 ± 10.8
	Inter	KNN	51.4 ± 3.2	50.9 ± 6.1	52.6 ± 3.1	51.7 ± 4.5	52.6 ± 3.7	52.5 ± 4.9	52.3 ± 3.8
		DT	50.5 ± 4.9	51.3 ± 7.7	51.2 ± 3.3	51.3 ± 6.7	51.3 ± 4.7	52.1 ± 7.8	51.6 ± 6.2
		AB	52.4 ± 5.5	54.0 ± 10.7	52.7 ± 3.2	52.2 ± 9.6	52.9 ± 5.2	53.4 ± 10.2	52.9 ± 9.1
		GNB	52.9 ± 4.7	53.2 ± 11.2	53.7 ± 2.3	52.6 ± 4.2	53.5 ± 3.5	54.3 ± 4.1	53.5 ± 3.3
		LDA	**54.2** **±** **5.3**	**57.3** **±** **11.9**	**54.4** **±** **3.8**	**54.4** **±** **5.8**	**55.3** **±** **5.3**	**56.0** **±** **10.1**	**55.1** **±** **5.8**
		QDA	53.1 ± 5.2	55.6 ± 12.1	53.9 ± 2.8	53.0 ± 4.8	53.2 ± 4.2	53.9 ± 5.9	52.6 ± 3.7
		SVM	52.1 ± 5.2	55.1 ± 12.5	53.9 ± 3.3	50.8 ± 5.4	54.5 ± 5.5	54.8 ± 9.2	53.1 ± 5.8
Ecological	Intra	KNN	**54.9** **±** **9.2**	56.9 ± 12.1	49.4 ± 6.1	**54.9** **±** **8.5**	49.6 ± 6.1	49.3 ± 6.2	49.6 ± 6.1
		DT	50.9 ± 8.1	55.9 ± 10.7	50.1 ± 5.9	52.7 ± 8.6	51.5 ± 8.9	54.0 ± 8.5	52.8 ± 8.6
		AB	52.7 ± 9.1	56.8 ± 11.9	49.7 ± 5.5	52.7 ± 8.7	52.3 ± 9.2	55.3 ± 9.8	53.2 ± 7.8
		GNB	52.8 ± 8.1	**59.7** **±** **12.6**	51.0 ± 5.4	53.3 ± 9.2	52.0 ± 8.6	54.5 ± 6.5	52.4 ± 8.3
		LDA	51.1 ± 8.3	56.9 ± 16.4	**51.9** **±** **8.9**	53.3 ± 10.9	**52.3** **±** **10.4**	54.2 ± 12.9	52.5 ± 10.5
		QDA	53.1 ± 7.6	57.8 ± 12.8	49.7 ± 5.5	53.0 ± 8.9	50.4 ± 6.0	52.4 ± 7.2	49.8 ± 5.7
		SVM	52.4 ± 10.3	59.4 ± 12.3	49.9 ± 5.9	54.2 ± 9.3	52.0 ± 9.5	**55.8** **±** **10.0**	**53.7** **±** **8.8**
	Inter	KNN	**54.4** **±** **7.3**	52.1 ± 9.0	52.7 ± 4.3	55.2 ± 6.6	**55.2** **±** **7.7**	51.5 ± 6.1	55.0 ± 5.7
		DT	50.8 ± 7.6	52.3 ± 12.7	50.5 ± 4.6	52.0 ± 7.9	50.7 ± 7.5	53.7 ± 8.7	53.1 ± 7.7
		AB	52.3 ± 6.1	55.9 ± 18.3	52.2 ± 4.3	53.8 ± 9.7	52.8 ± 6.5	54.4 ± 15.4	53.7 ± 10.3
		GNB	52.3 ± 4.2	54.3 ± 16.0	51.9 ± 4.1	51.4 ± 3.7	53.6 ± 4.8	53.8 ± 4.1	52.8 ± 4.5
		LDA	52.6 ± 5.5	**59.8** **±** **18.6**	53.0 ± 4.5	**55.3** **±** **9.1**	54.3 ± 6.1	**56.5** **±** **12.9**	**56.1** **±** **8.7**
		QDA	52.5 ± 4.4	58.3 ± 17.6	**53.3** **±** **5.4**	52.8 ± 4.5	53.4 ± 4.9	52.9 ± 7.0	53.3 ± 5.3
		SVM	52.6 ± 5.9	57.2 ± 18.8	52.1 ± 5.1	54.5 ± 7.6	53.8 ± 6.4	53.5 ± 10.7	55.6 ± 8.7

*Bold: Best accuracy per feature type and validation method*.

## 4. Discussion

The main objectives of this study were: (i) to characterize and estimate mental workload in a MUM-T setting with a search and rescue mission scenario; (ii) to assess the impact of the validation procedure on classification accuracy by comparing the results obtained for the traditional and the ecological methods. To achieve these objectives, an important amount of developments and research, ranging from different kinds of interactions between humans and UAVs, different types of workload experienced by human agents, and about how to alter mental workload without loosing realism of a MUM-T mission has been conducted to create a setup that resembles all the working principles of a MUM-T operation.

The subjective and behavioral results have enabled us to confirm the ability of the implemented missions to elicit two levels of mental workload. Indeed, in the high load condition participants' performance significantly decreased and they reported a higher load than in the low load condition, which is in accordance with the literature (Taylor et al., 2005; Bruggen, 2015; Gateau et al., 2015, 2018). It appeared after hand that some participants translated the English ATC commands into their native language for memorization, an additional processing step that has most certainly affected their performance. However participants' strategies were unfortunately not systematically recorded and could not be analyzed. It might also have been particularly interesting to evaluate their adherence to the given priorities. Indeed, immediately after ATC commands, participants could make their priorities wrong. Here the participants had to choose between shifting to the new heading first or first putting the radio values for UAVs. In this case the longer they delayed the heading, the worst the score would be. For the next step—implementing real time mental state estimation and system adaptation—the design of a composite score with weights applied on the performance scores corresponding to the priorities will be required by the planning algorithm.

Interestingly, in addition to the subjective and behavioral results, the physiological measurements were also in accordance with the literature with a significant increase in heart rate and decrease in heart rate variability (Heard et al., 2018), as well as a significant increase in power in the EEG *β* and *γ* bands and an increase of the engagement ratio with an increase in mental workload. These power modulations are in accordance with the literature and have been linked to modifications in the alertness level, integration of sensory inputs, and working memory load (Pope et al., 1995; Kumar and Bhuvaneswari, 2012; Borghini et al., 2014). Moreover, as expected from the literature (Borghini et al., 2014; Roy et al., 2016b), there was a decrease in alpha power at parietal sites, however this effect did not reach significance. Concerning the results obtained for ET features, pupil dilation increased with an increase in mental workload as expected (Heard et al., 2018). Moreover, fixation duration decreased with an increase in mental workload which reflects the more demanding the scenario was on attentional resources that had to be allocated to several tasks.

A thorough benchmarking of several feature combinations and classifiers was performed in order to determine an efficient mental workload estimation pipeline for this MUM-T scenario. Moreover, in order to assess the impact of the validation procedure, due to time-related non-stationarity effects on the physiological signals, different classification designs were studied and implemented. In particular, an ecological design was proposed to better reflect a real world process by training the estimation pipeline on data acquired beforehand (either from previous subjects or data from the beginning of the session) and testing the pipeline on new data (either from a new subject or from data of the end of the session). This validation design was compared to a traditional one. As was hypothesized based on the literature (Roy et al., 2013, 2016b), mental workload estimation dropped significantly when using the ecological design, for the intra-subject classification. Note that this is also the case when systems are trained in a controlled setup and then evaluated in a real world setup. However, this accuracy performance decrease should not be seen as a limitation. Rather it should be taken as an opportunity to work on further developments in order to exploit new promising methods for such operational scenarios. For instance, transfer learning, automatic feature selection, deep learning, and ensemble learning could be good options to look forward. Fahimi et al. proposed and evaluated a transfer learning technique based on Deep Neural Networks, in which a classifier is trained with a *leave-one-out* procedure and then further retrained using a small sample set of test subject's data (Fahimi et al., 2019). However, they did not consider the signal's non-stationarity, which could bring different results in a more realistic scenario. Whereas in this study a basic form of transfer learning was implemented with the inter-subject ecological classification design which took into consideration the non-stationarity effect of EEG.

In light of these results, it appears that HR and HRV features from ECG, pupil dilation feature from ET, and beta, gamma, and engagement index features from EEG could possibly be exploited in a MUM-T scenario in order to estimate human mental workload through classification means. In addition regarding the EEG features, specific frontal, temporal and parieto-occipital sites have been identified that could lead to better mental workload estimation results, i.e., Fp2, FC5, FC6, T7, T8, CP5, P8, O1, Oz, and O2. More specifically, regarding the features that are most useful for mental workload assessment in our scenarios, it stood out that ECG features provided the best results compared to EEG and ET ones. One of the reasons could be the number of features, only 2 ECG features were used for classification, compared to 60 for EEG and 16 for ET. Therefore, automatic feature selection as proposed by Climente-González et al. (2019), could also be another promising research venue to look into while designing a better estimation pipeline for real world mental workload monitoring applications. Finally, concerning EEG features, one could also look into more robust features to avoid non-stationarity effects, such as ERPs as proposed by Roy et al. (2016b). Besides feature combinations, several classifiers were benchmarked in this study. The ones that gave the best results were the Adaboost, the Linear Discriminant Analysis and the Support Vector Machine. Although classifiers based on Riemannian geometry are known to give outstanding results for cognitive state estimation based on EEG data (Appriou et al., 2020), we could not use them for features others than EEG, and therefore they were not included in our benchmark. However they should be investigated in combination with others for peripheral signals in order to improve the mental workload estimation. Also, signal conditioning methods should be considered and benchmarked, such as spatial filtering methods (Roy et al., 2015).

Some of the limitations of this work that should be considered for future studies could concern a more accurate chance level adjustment by considering the number of features in addition to the number of trials per class (Combrisson and Jerbi, 2015). Another limitation to be addressed would be the need to evaluate the benefit of transfer learning methods to overcome the time-related non-stationarity issue. Moreover, these results need to be further validated by increasing the number of participants in order to increase the amount of data and robustness of the results, but also by either eliminating artifact sources other than ocular ones, which might have affected the gamma range, or by only focusing on lower frequency bands, as well as by evaluating the benefit of performing and individualized frequency band computation. Moreover, a necessary step in future works consists in working on the implementation of additional methods such as Riemmanian methods that could be employed to fuse all physiological recordings (Jiang et al., 2019), as well as thoroughly evaluating the potential use of data demanding algorithms such as the ones based on deep learning (Schirrmeister et al., 2017; Chakladar et al., 2020).

In the near future, this work will be extended towards the completion of a whole pBCI pipeline as the one showed in Figure 4. The pipeline output could be seen as the output of a monitoring system. This monitoring system could then feed an appropriated sequential decision-making system (e.g., planner) that would take the estimated state of both types of agents: the artificial ones, and the human pilot, and then, choose the most appropriate agent, given its skills and current capabilities, for performing a given task at hand. Hence, work will have to be performed regarding the most appropriate ways to mitigate operator mental states, such as task allocation, i.e., adaptive automation human-computer interface studies (Aricò et al., 2016; Di Flumeri et al., 2019). A solution to perform this task allocation is to use sequential-decision making. A sequential-decision making model able to consider uncertainties, such as monitoring system outputs' inaccuracy, while taking into consideration long-term mission goals, would be a promising candidate. Moreover, the partial observability of the human pilot state and the low accuracy of the monitoring system (e.g., classifier) could be also compensated by such an appropriate sequential decision-making framework. Partial Observable Markov Decision Process seems to be an excellent long-term decision framework to implement such an adaptive symbiotic teaming (Chanel et al., 2020b; Roy et al., 2020).

## 5. Conclusion

This research work has provided a Manned-Unmanned Teaming (MUM-T) environment that aims to mimic a real world search and rescue operation, while providing a way to characterize and estimate the mental workload of a human agent based on subjective, behavioral and physiological measures. Cardiac measures and the Adaboost, Linear Discriminant Analysis and Support Vector Machine classifiers gave the best mental workload estimation performance. Furthermore, an ecological classification validation design was proposed and evaluated. The negative effect of the time-related non-stationarity of physiological data (e.g., EEG features) on classification performance was statistically assessed and the authors believe it should be considered when developing solutions for real world applications. In conclusion, this work paves the way towards an interaction control system design that could take decisions and assign tasks based on the state of artificial agents and also based on human mental state estimation. The remaining part is to complete the bigger picture of this pBCI system by thoroughly considering inputs from all involved agents, current state and goals of the mission; and then, taking decisions that could enhance the mission performance while keeping both the agents in safer and best productive state.

## Data Availability Statement

The datasets presented in this article are not readily available because not shareable. Requests to access the datasets should be directed to gaganpreet.11@gmail.com.

## Ethics Statement

The studies involving human participants were reviewed and approved by CER Toulouse, Id number: 2019-137. The patients/participants provided their written informed consent to participate in this study.

## Author Contributions

GS, CC, and RR: study conception and design, data analysis and interpretation, and writing the article. GS: protocol implementation and data acquisition. All authors have approved the current manuscript.

## Conflict of Interest

The authors declare that the research was conducted in the absence of any commercial or financial relationships that could be construed as a potential conflict of interest.

## Publisher's Note

All claims expressed in this article are solely those of the authors and do not necessarily represent those of their affiliated organizations, or those of the publisher, the editors and the reviewers. Any product that may be evaluated in this article, or claim that may be made by its manufacturer, is not guaranteed or endorsed by the publisher.

## Footnotes

^1^ https://www.aerofly.com/

^2^LabStreamingLayer (LSL) library version 1.13, see documentation at https://labstreaminglayer.readthedocs.io/index.html.

^3^MNE version 0.22.1, see documentation at https://mne.tools/stable/index.html

^4^see https://github.com/Aura-healthcare/hrv-analysis.
